# Structure Based Modeling of Small Molecules Binding to the TLR7 by Atomistic Level Simulations

**DOI:** 10.3390/molecules20058316

**Published:** 2015-05-08

**Authors:** Francesco Gentile, Marco A. Deriu, Ginevra Licandro, Alessio Prunotto, Andrea Danani, Jack A. Tuszynski

**Affiliations:** 1 Department of Physics, University of Alberta, Edmonton, AB T6G 2E1, Canada; E-Mail: fgentile@ualberta.ca; 2 Institute of Computer Integrated Manufacturing for Sustainable Innovation, Department of Innovative Technologies, University of Applied Sciences and Arts of Southern Switzerland (SUPSI), Manno CH-6928, Switzerland; E-Mails: marco.deriu@supsi.ch (M.A.D.); ginevra.licandro@icimsi.ch (G.L.); alessio.prunotto@supsi.ch (A.P.); andrea.danani@supsi.ch (A.D.); 3 Cross Cancer Institute, Department of Oncology, University of Alberta, Edmonton, AB T6G 1Z2, Canada

**Keywords:** toll-like receptors, molecular docking, homology modeling, molecular dynamics, imidazoquinoline, immune system, adenine derivatives

## Abstract

Toll-Like Receptors (TLR) are a large family of proteins involved in the immune system response. Both the activation and the inhibition of these receptors can have positive effects on several diseases, including viral pathologies and cancer, therefore prompting the development of new compounds. In order to provide new indications for the design of Toll-Like Receptor 7 (TLR7)-targeting drugs, the mechanism of interaction between the TLR7 and two important classes of agonists (imidazoquinoline and adenine derivatives) was investigated through docking and Molecular Dynamics simulations. To perform the computational analysis, a new model for the dimeric form of the receptors was necessary and therefore created. Qualitative and quantitative differences between agonists and inactive compounds were determined. The *in silico* results were compared with previous experimental observations and employed to define the ligand binding mechanism of TLR7.

## 1. Introduction

The human immune system has been traditionally divided into the innate immune system and the adaptive immune system. The innate immune system’s main function is to recognize pathogen-associated molecular patterns (PAMPs) [1]. This process allows the immune system to distinguish self from non-self. The PAMPs recognized by the innate immune system are fixed in the genome, whereas in the adaptive immune system new receptors are acquired following antigen exposure. In a healthy person the innate and adaptive immune systems work together and mount a defensive response to infection. Toll-like receptors (TLRs) are an important class of pattern recognition receptors [1]. The PAMPs recognized by TLRs include bacterial cell wall components, other bacteria-specific molecules, and viral double-stranded RNA. Each TLR has structural differences and specificity to particular PAMPs. Generally, TLRs are transmembrane proteins localized at the plasma membrane, except for TLR7, TLR8, and TLR9, which are localized at endosomal membranes [1]. Some TLRs, such as TLR7, must first form dimers or heterodimers in order to trigger their response to PAMP binding. There is still little information regarding the molecular mechanism by which various PAMPs interact with the corresponding TLRs. Our interest has been focused on TLR7, which has been shown to recognize single-stranded viral RNA [2], and is also the target of a number of investigational therapeutic agents [3]. TLR7 is part of a subfamily of TLRs which localize at intracellular endosomes (along with TLR8 and TLR9). Drugs targeting TLRs could be useful in treating a number of disease conditions, for example specific and selective agonists of TLRs could be useful in the treatment of certain infections.

Toll-Like Receptor 7 (TLR7) is a transmembrane protein localized within the endosomal compartment. It is mainly expressed in lung, brain, stomach, placenta, and peripheral blood mononuclear cells (PBMCs), such as dendritic cells, monocytes, macrophages and B-lymphocytes. This receptor is involved in the innate immune system response [1] and naturally senses viral single-stranded RNA such as Influenza [2] and Coxsackie B [4] viruses, representing a pivotal receptor for the immune system activation [5]. Moreover, the interferon (IFN)-mediated innate immune response, which is related to the TLR7 activation, has been shown to be interfered with by the Hepatitis C virus [6]. It was recently demonstrated that it is also involved in self-nucleic acid recognition, gaining therapeutic interest for the treatment of a huge number of inflammatory diseases [5,7]. Furthermore, a relation between TLR7 and cancer has been also lately described [8,9]; indeed the receptor is highly expressed in cancer cells such as chronic lymphocytic leukemia cells [10], and it regulates the pancreatic carcinogenesis in humans and mice [11]. Interestingly, several small molecules activating TLR7 have showed antitumoral activities [3].

In humans the protein is composed of 1049 amino acids, divided into an ectodomain of twenty seven leucine-rich repeat (LRR) regions, a trans-membrane domain (TM) and a cytosolic Toll/interleukin-1 (IL-1) receptor (TIR) domain [12]. The ectodomain is localized into the endosome, exposed to variable acid pH [13]. The activation of TLR7 is strongly related to a proteolytic maturation, internally the zone between LLR14 and 15, within several putative sites for the proteolytic cleavage [13,14]. Moreover, the N-terminal removal is essential for the signaling but not for ligand binding [15]; thus, a re-association process similar to the one present in Toll-like Receptor 8 (TLR8) [16] is likely to occur [17,18,19].

The huge number of pathologies in which the TLR7 is involved has led to an increasing interest in developing new drugs able to bind with this protein; both the activation and the inhibition of the receptor could represent a fruitful therapeutic approach, depending on the disease characteristics. The activation can enhance the immune response to viral agents and cancer, while the inhibition of the receptor can be used in treatments of some chronic inflammatory diseases [20]. Imidazoquinoline derivatives are a family of tricyclic organic molecules that are widely used in the targeting of TLR7. These compounds have shown powerful antiviral and antitumoral activity [19,21,22]. Imiquimod (R-837) [23] is an imidazoquinoline derivative approved by the Federal Drug Administration (FDA) for the treatment of viral diseases, skin cancer and metastasis [7,24,25,26,27]. Imiquimod is provided as topical treatment and activates TLR7, but not TLR8 [28]. Resiquimod (R-848) is a dual agonist for TLR7 and TLR8, showing an Imiquimod-like effect on the immune response [29]; R-848 activity is reported in terms of EC_50_ as 607 nM with a 240 nM standard deviation [30]. CL097 is another imidazoquinoline derivative that activates both the receptors [17]. These compounds are immunomodulatory molecules that induce the production of several cytokines, such as IFN-α, TFN-α, and IL-6. Nucleoside analogs constitute another class of ligands for TLR7 [28,31,32,33], derived from the bases of nucleic acids. Forsbach *et al.* have generated variants of the compound 52455 (Sumitomo) by altering the pyrimidine/imidazole ring system or side group exchanges; the resulting small molecules are the 52455, 52457, 52459, 52763, 52542, and 52587; three of these compounds showed an effect on TLR7 in activating nuclear factor-kappaB (NF-κB) on HEK293 cells: compound 52455 showed an EC_50_ of 103 nM with a standard deviation of 12 nM, compound 52542 showed 353 nM with a standard deviation of 96 nM and compound 52763 showed 965 nM with a 283 nM standard deviation; the other compounds did not show any activity on TLR7 [30]. 1V209 [34] is another TLR7 agonist with a structure similar to 52455 and 52542.

Computational methods have been demonstrated to be powerful tools to study the behavior of proteins and small molecules at the atomistic scale. A pivotal requirement for computational analysis involving receptor studies is a reliable three dimensional structure of the protein. Homology modeling technique has become an important tool in drug design and discovery [35], as it allows to build a realistic *in silico* model, wherever an experimental (X-ray or NMR) one is not available (as the case of TLR7). It refers to constructing an atomic-resolution model of the target protein starting from the alignment of the amino acid sequence with the one of a related homologous (the template) having a defined experimental (crystallographic) three-dimensional (3D) structure. It is well accepted that the method can produce reliable models only if the similarity in amino acid chains between template and target is higher than 30%, with few exceptions [36]. Recently, at least four models of the receptor were published. Wei *et al.* produced a model for the TLR7 ectodomain, identifying putative binding residues [37]. The same author then published homology models for several TLR family proteins, including a newer one for TLR7 [38]. Yu *et al.* homology modeling of TLR7 resulted in a prediction of the dimeric structure and the production of a pharmacophore [39]. Recently, the model from Tseng *et al.* was used in molecular docking experiments, assessing the residues involved in ligand binding. Furthermore putative dimeric structures were predicted and interestingly the most probable one was found to be very similar to TLR8 X-ray dimeric structure [16]. It is worth mentioning that all the models were built by using as templates other TLR structures, but not TLR8 because of the crystal structure unavailability.

In order to better understand the mechanism of activation of the receptor by small molecules, and thus lay the basis for the development of novel therapeutic compounds, we built an all-atom model of the dimeric structure of the TLR7 based on a recently published TLR8 template, which includes two molecules of a dual agonist. This allowed us to obtain a realistic model also for the binding zone conformation, and nine compounds from two classes of known agonists were docked. Once the binding positions had been disclosed, we ran Molecular Dynamics simulations in order to equilibrate the complexes; this was made in order to introduce the full flexibility of the receptor, which was completely neglected during the docking procedure. Finally, to obtain a reliable energetic rank, the Molecular Mechanics Generalized Born Surface Area (MMGBSA) technique was employed, to verify the capability of our model to distinguish between TLR7 agonists and compounds that are unable to activate the receptor; in this manner, the weakness of the docking scoring function could be overcome. Moreover, this approach furnished quantitative indication about the importance of each residue in the binding zone on agonist recognition and interaction.

## 2. Results and Discussion

### 2.1. Homology Modeling

**Figure 1:**
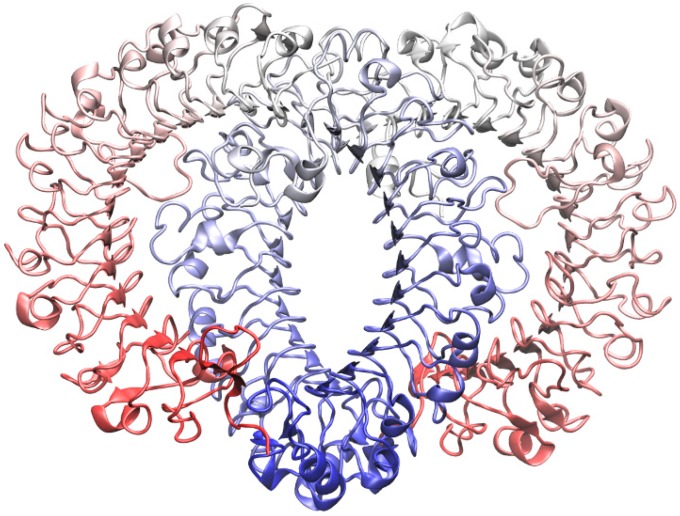
The new model for the Toll-Like Receptor 7 (TLR7) dimer obtained with homology modeling, using the crystallographic structure of TLR8 as template. In both the monomers the respective C-terminals are represented in blue while N-terminals are in red.

The TLR8 endosomal domain consists of 817 residues for each monomer; residues from 434–458 are missed after the proteolytic cleavage; in TLR7, residues from 436–478 were deleted in each chain during the alignment procedure, as Asn^479^ is one of the probable sites of cleavage [13]. The sequence identity between the ectodomains of the two receptors was approximately 46%, including a series of highly conserved residues in the binding zone: Asp^543^, Gly^572^, Thr^574^ in one monomer and Phe^346^, Tyr^353^, Gly^376^, Val^378^, and Phe^405^ in the other one for TLR8; in TLR7 the respective residues are Asp^555^, Gly^584^, Thr^586^, Phe^349^, Tyr^356^, Gly^379^, Val^381^, and Phe^408^; the modeling of the receptor resulted in 754 residues. The new TLR7 model is reported in Figure 1. The results from the structural evaluation are reported in Table 1: the ERRAT scores for the holo-structure (before and after the minimization) are comparable with other TLR7 models [39]. The PROCHECK results indicate a small number of amino acids that present a wrong value for the Ψ and Φ angles (1%), and a large number of residues with torsion values in the theoretically correct part of the Ramachandran plot (70.9% after the optimization); these values are comparable with other TLR7 homology models, as for example the percentage of amino acids in the most favorable region of the plot was found around a value of 68% for Wei’s second model [38] and 72% for Tseng’s model [40], while the percentage of residues in the most favorable and additional regions was 97.4% in the first Wei’s model [37]. The optimization process performed in pmemd.cuda [41,42,43] permits a slight improvement in the structural quality of our TLR7 model. A small number of dislocated angles were detected also in the TLR8 experimental structure.

**Table 1:** Structural evaluations performed for the Toll-Like Receptor 8 (TLR8) crystallographic structure, the TLR7 model obtained with homology modeling and its minimized form.

Structure	ERRAT Quality Factor	Procheck			
		Core	Additional	Generously	Disallowed
TLR8	84.364	79.0	20.1	0.5	0.4
TLR7	73.861	69.0	28.1	1.9	1.0
Minimized TLR7 ^1^	76.506	70.9	26.1	2.0	1.0

ERRAT results refer to the percentage of the protein for which the calculated error value falls below the 95% rejection limit. PROCHECK results refer to the percentage of angles lying in the relative zones of Ramachandran plot; ^1^ TLR7 structure minimized for the docking (keeping restrained both the protein backbone and heavy atoms of CL097), see text for more details.

### 2.2. Molecular Docking

**Figure 2:**
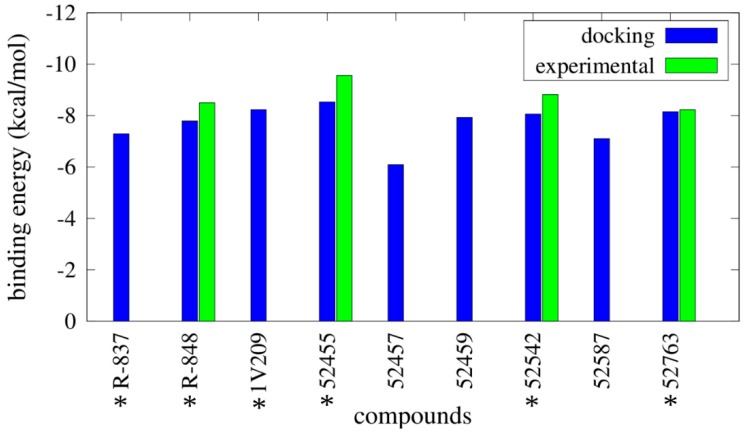
Binding energies for the best conformations obtained from docking simulations performed with Autodock. Computational binding energies (in blue) are compared with the available experimental binding energies (in green), calculated as RTlnEC_50_. * indicates the TLR7 agonists.

**Figure 3:**
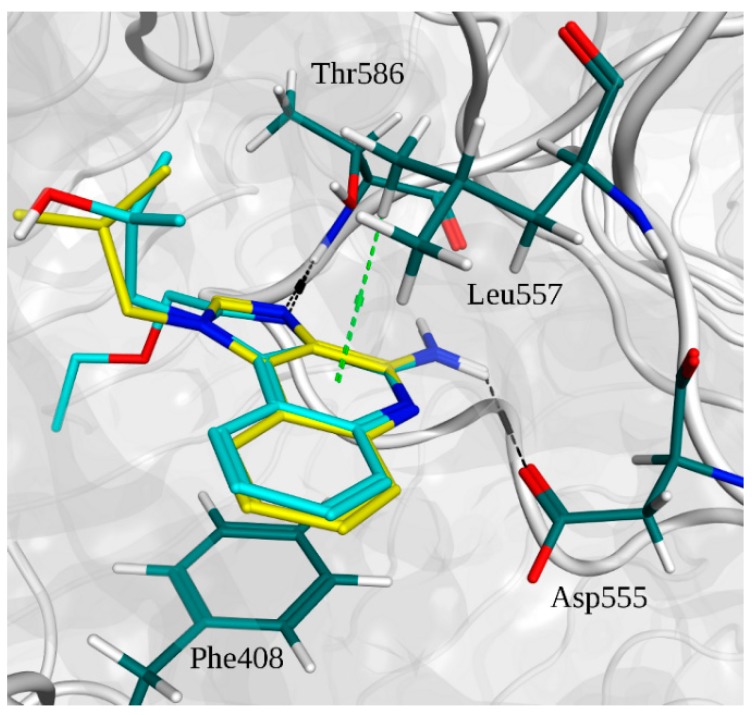
Superimposition of the binding poses of imidazoquinoline derivatives and main interactions with TLR7. R-837 carbons are depicted in yellow. R-848 carbons are depicted in cyan. Residue carbons are depicted in dark green. Nitrogens are depicted in blue. Polar hydrogens are depicted in white. Oxygens are depicted in red. Pocket surface is represented in gray. Hydrogen bonds are represented in black. C-H-π interactions are in light green.

Binding energy results from Autodock are reported in Figure 2 (and Table S1), as well as the experimental binding energies. It was clearly difficult to distinguish between agonists and not agonists based on the obtained energies, as all the compounds show binding energies between −6.09 kcal/mol and −8.53 kcal/mol. Imidazoquinoline derivatives share a similar binding pose within the pocket (Figure 3).

**Figure 4:**
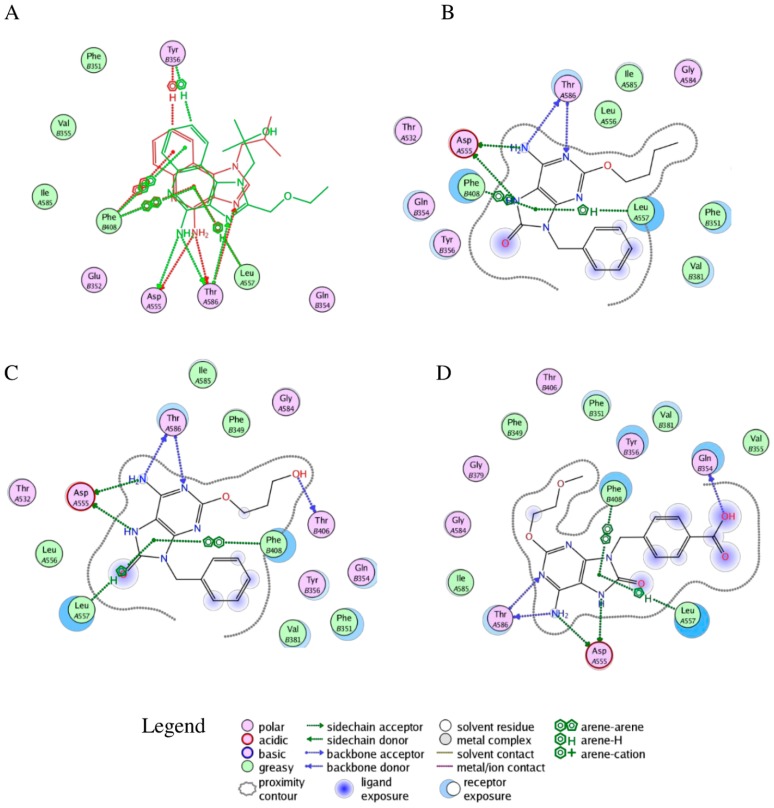
Interactions between selected agonists and TLR7. (**A**) Superimposition of R-837 (red) and R-848 (green) binding poses; (**B**) Binding pose for compound 52455; (**C**) Binding pose for compound 52452; (**D**) Binding pose for compound 1V209. A and B suffixes on the residues refer to the relative TLR7 monomers.

**Figure 5:**
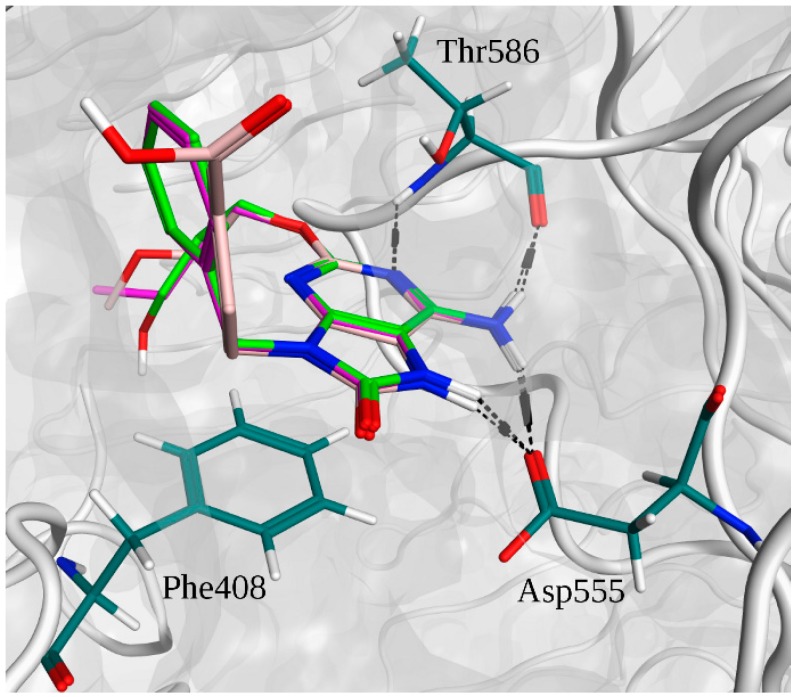
Superimposition of the binding poses of adenine derivative agonists and main interactions with TLR7.The 52455 carbons are depicted in purple. The 52542 carbons are depicted in light green. The 1V209 carbons are depicted in pink. Residue carbons are depicted in dark green. Nitrogens are depicted in blue. Polar hydrogens are depicted in white. Oxygens are depicted in red. Pocket surface is represented in gray. Hydrogen bonds are represented in black.

In Figure 4A, the superimpositions of MOE interaction diagrams of R-837 and R-848 with TLR7 are reported. The interactions with imidazoquinoline derivatives are mainly established through highly conserved residues of TLR7 and 8, such as Asp^543/555^ and Thr^574/586^ which participate with three hydrogen bonds in the binding with the two ligands. However, in TLR7 the C-H-π interaction between Leu^557^ and one of the aromatic rings is also present; it is worth highlighting that this residue is not conserved in TLR8 and that Leu^557^ has been already described as one of the most important residues for the ligand recognition in TLR7 [40]. This finding, in accordance with the published results, could explain both the reduced activity of the R-848 and the absence of the activity of the R-837 on TLR8 [16]. The 52X series and the 1V209 were docked in the same binding pocket of the TLR7; a similar way to interact with the target was found for 52455, 52542 and 1V209 (Figure 5). Two hydrogen bonds are established between the molecules and residues Asp^555^, and two with Thr^586^, whereas Phe^408^ and Leu^557^ interact with the small molecules via arene-arene and C-H-π interactions, respectively (Figure 4B–D).

### 2.3. Molecular Dynamics Simulations

**Figure 6:**
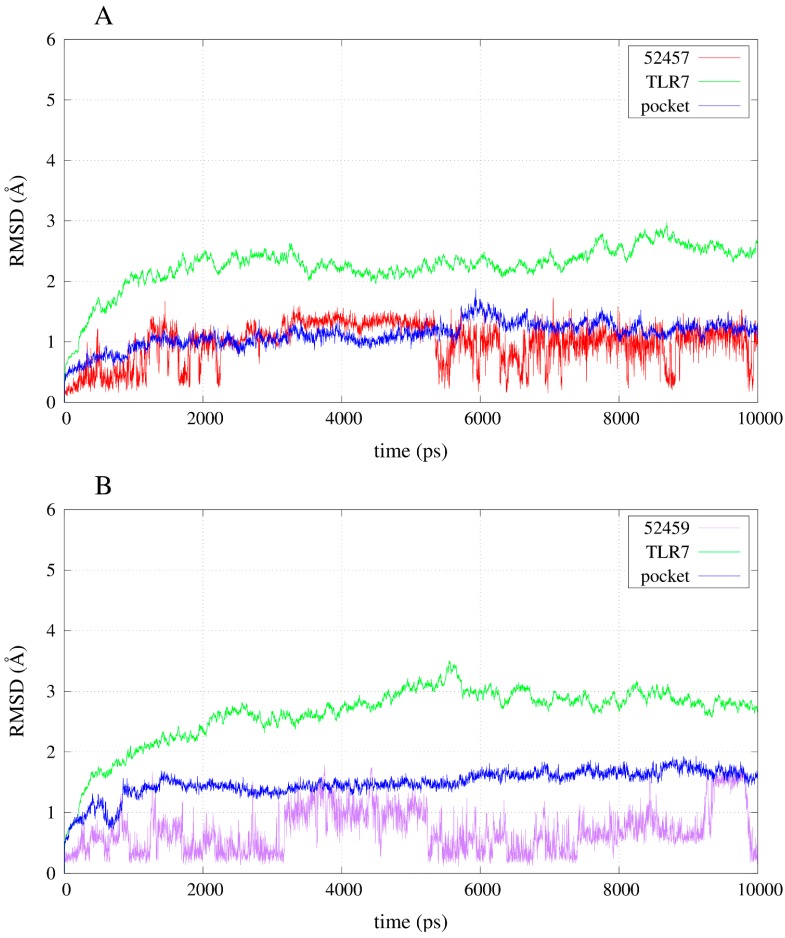
RMSD trends for backbone, binding pocket and two selected inactive compounds. (**A**) RMSD for 52457 in complex with TLR7; (**B**) RMSD for 52459 in complex with TLR7.

**Figure 7:**
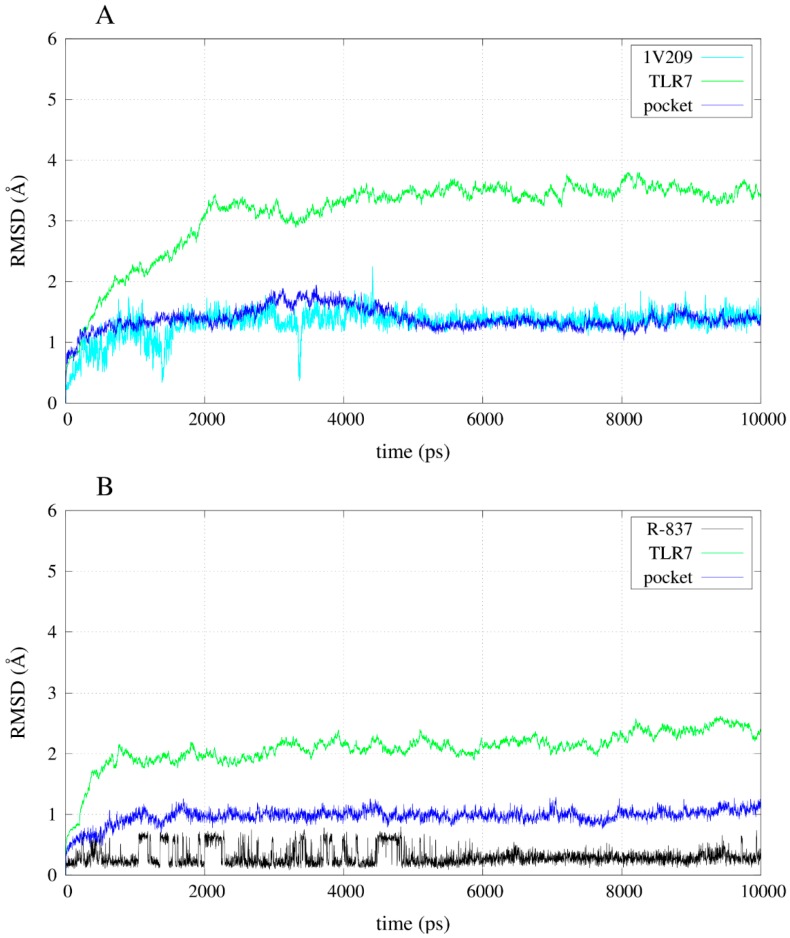
RMSD trends for backbone, binding pocket and two selected agonists. (**A**) RMSD for 1V209 in complex with TLR7; (**B**) RMSD for R-837 in complex with TLR7.

The simulation of the apo-structure allowed the observation of an RMSD (root mean square deviation) trend that reached a plateau around 3 Å (Figure S1). Moreover, the total energy of the system became stable after the instability due to the heating and the release of the harmonic restraints (Figure S2). The RMSD trends of some of the compounds during the simulations allow the observation that the instability of the poses for the inactives compared with the agonists: 52459 and 52457 fluctuated a lot during the simulations (Figure 6), whereas R-837 and 1V209 showed a stable RMSD trend (Figure 7). All the complexes show a relative stable RMSD for the protein after the equilibration, with a difference between about 2.5 and 3.5 Å from the starting structure; similar to the one of the equilibrated apo-structure. The binding pocket is roughly equilibrated in every case, with values of difference with the original conformation between 1 and 2 Å.

### 2.4. Free Energy Estimates

**Figure 8:**
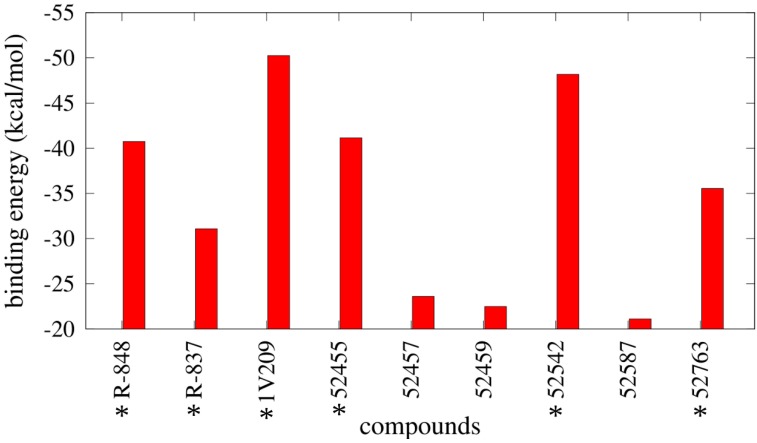
Binding energies derived from Molecular Mechanics Generalized Born Surface Area (MMGBSA) calculations. * indicates the TLR7 agonists.

**Figure 9:**
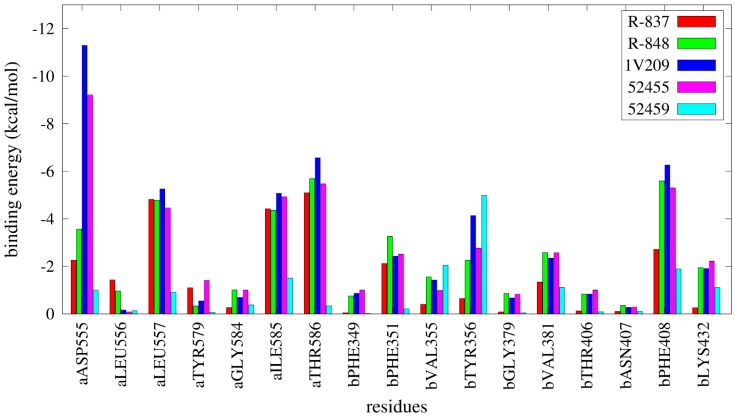
Pairwise per-residue decomposition of binding energies for selected agonists and one inactive compound. a and b residue suffixes refer to the relative TLR7 monomer.

The results from the MMGBSA calculations are reported in Figure 8 and Table S2 and show a notable difference in binding energies between agonists and inactive compounds, as 52459, 52457, and 52587 have relative unfavorable binding energies compared with the other compounds; specifically, 1V209 showed the best binding energy toward the set. Regarding the pairwise decomposition of the binding energy, interactions of residues that are within 5 Å from the docked pose of the compounds were evaluated. In Figure 9 (and Table S3), a comparison between four agonists (R-837, R-848, 1V209 and 52455) and one non-agonist (52459) is reported. As expected, the principal differences are present in the two most important residues for ligand matching: Asp^555^ and Thr^586^; the agonists show a considerable contribution from these two residues, in particular Asp^555^ is the largest contributor for 1V209 and 52455, as expected by its similarities in chemical structure; in the case of both R-837 and R-848 it interacts with only one hydrogen bond. Regarding the 52459, the energetic contributions from Asp^555^ and Thr^586^ are negligible. It is worth mentioning that residues from the other chain played also an important role in receptor-ligand interactions, especially Phe^408^ was able to establish a hydrophobic interaction with all the ligands.

## 3. Discussion

The work presented in this paper is a computational study focused on the activation of TLR7 dimer formation using several known agonists. It deals with known activators of TLR7 whose biological action has been previously investigated but no details of molecular action on the target have been given before. This is not intended to be a virtual screening effort since we knew which compounds already showed the desired activity. Our work elucidated their detailed mode of action at an atomistic level.

In this work, we proposed a novel structural model for the dimeric form of the Toll-like receptor 7. The lack of an experimental three-dimensional structure constituted an obstacle for using computational tools in order to accurately investigate the behavior of ligands within the protein. Although other homology models were published before for TLR7, we propose a model based on the recently disclosed TLR8 dimeric structure as template, which represents the most similar protein in terms of ligands, functions, cellular localization, and sequence similarity. Our structural evaluation confirms a good overall quality of this model, and a series of known ligands and inactive molecules were docked in the putative binding pocket around residue Asp^555^. Molecular modeling techniques were employed to find on our TLR7 model possible orientations of imidazoquinoline and adenine derivatives that work as activators of TLR7. Binding affinities calculated over the simulations allowed us to obtain a relative rank between compounds that was consistent with the experimental data about the activities; 1V209 demonstrated the best binding energy and agonists showed stronger binding energies compared to the inactive ones; taking into account the absence of activation of NF-κB and the less favorable binding energy of the 52457, 52459 and 52587 compounds observed *in silico*, we could hypothesize an extremely reduced affinity and incapability to bind with the target. These data not only confirmed our model as a powerful platform to find new candidates, but also highlighted a direct correlation between binding energy and agonist activity. It is worth mentioning that the described binding energies, obtained with the MMGBSA method, do not include the entropic contribution, so they are relative binding energies; however, the discussed results are not affected because our objective was to verify if the presented model was able to distinguish between agonists and inactive molecules. Finally, the importance of the residues in the binding pocket in terms of ligand binding contribution was assessed by a quantitative measure of the decomposition of free energies. Particularly, Asp^555^, Ile^585^, Thr^586^, and Phe^408^ were found to be the strongest contributors in agonist recognition; in addition Leu^557^ was established to be also important for the ligand recognition. This latter observation, and the related C-H-π interaction, could confirm the hypothesis of Tanji *et al.* [16] and explain the selectivity of the ligands for the TLR7 (as the residue is not conserved in the TLR8). From our results it is quite clear that the docking technique implemented in Autodock is a powerful tool to be used in order to rapidly and efficiently place a ligand in the best possible conformation within the protein binding site; however, this technique suffers from several issues, mainly related with the rigidity of the receptor and the cheapness of the scoring function during the simulation, that in fact limits the power of the software in distinguishing between real and false positive results; our workflow permits us to overcome these limits by post-processing the binding poses using MD simulations and using more sophisticated scoring functions, respectively. In conclusion, our work not only produced a high quality platform to perform virtual screening on possible TLR7 agonists or antagonist by using a complete, reliable protocol, but it also disclosed the molecular basis for the recognition of two classes of major agonists, namely imidazoquinoline and adenine derivatives, leading to further optimization of new derivatives.

## 4. Experimental Section

In this work, different molecular modeling approaches were sequentially employed to investigate interactions between several small molecules and the TLR7 receptor. Homology modeling techniques were employed to obtain an all-atom structure of the protein, using as template one of the crystallographic structure of TLR8 [16]. The structure was refined with a minimization process in explicit solvent, then molecular docking of small molecules was performed in order to place the molecules into the binding pocket. Further Molecular Dynamics simulations were performed for the complexes, and the MMGBSA technique was finally used to obtain an average of the relative binding energies.

### 4.1. Homology Modeling

The structure of TLR8 containing two co-crystallized molecules of CL097 (Protein Data Bank id: 3W3J), an agonist of both TLR7 and TLR8 [17], was chosen as template for the homology modeling process performed with Molecular Operating Environment software (MOE) [44]. The amino acid sequences of TLR7 and TLR8 were aligned using the A-star algorithm [45] in MOE-Align [46]. The two molecules of CL097 were kept conserved during the building of the new model, as MOE homology modeling routine allows fixed small molecules to be kept in the same position during the building of a new structure, thus permitting the shape of the binding pocket to be maintained in the final structure Ten different protein structures were obtained and ranked by calculating the electrostatic energy of solvation with the General Born Integration Volume (GB/VI) method [47] using *ff12SB* force field parameters for the protein [48] and EHT parameters for the ligands [49]. The best ranked model was chosen as final model which was then refined in MOE using 500 steps of Conjugate Gradients minimization, the parameters of the ff12SB force field for the protein [48] and ETH (Extended Hückel Theory) parameters for the ligands [49]. The protonation states were assigned at pH 5.5 using MOE Protonate 3D [50].

A successive stage of minimization was performed using Amber12 pmemd.cuda with the goal of optimizing the shape of the binding pocket. Missing parameters for the small molecules were assigned using AmberTools12 antechamber [51]; ff12SB [48]and GAFF [52] parameters were assigned for the protein and for the ligands respectively, with AmberTools12 tleap [41]. The system was inserted into an octahedral box with a buffer of 12 Å of TIP3P water molecules [53] and neutralized with chlorine ions. The cut-off for the long range interactions was set to 9 Å. One thousand steps of Steepest Descent method, followed by 1000 steps of Conjugate Gradients, were performed in Amber12 pmemd. cuda [41,42,43], applying a restraint of 500 kcal/((mol)(Å^2^)) to the protein. Then we ran 2000 Steepest Descent steps, followed by 3000 Conjugate Gradients steps, reducing the restraint force to 4 kcal/mol/Å^2^ applied to the protein backbone and heavy atoms of the ligands only. The structural evaluation of the optimized model was then performed using ERRAT [54], which evaluates the statistics of the pairwise non-covalently bonded interactions between heavy atoms, and PROCHECK [55], which provides information regarding the percentage of amino acids in allowed/disallowed regions of the Ramachandran plot.

### 4.2. Molecular Docking

**Figure 10:**
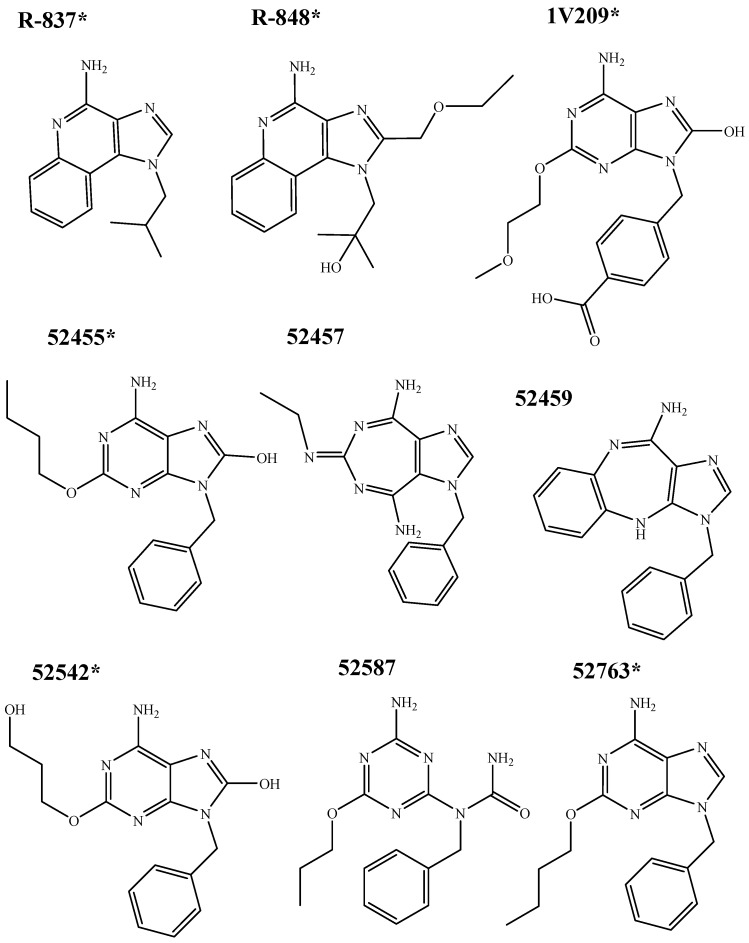
Chemical structures of tested imidazoquinoline and adenine derivatives. * indicates the TLR7 agonists.

Nine compounds were docked, including two imidazoquinoline derivatives (R-837 and R-848), and the 52X series, divided into four adenine derivative agonists (1V209, 52455, 52542 and 52763) and three inactive adenine derivatives (52457, 52459 and 52587), intended as unable to trigger the activation of the NF-κB. The structures of R-837 an R-848 were taken from PubChem [56] (CID 57469 and 159603, respectively); adenine derivatives structures were designed using ChemDraw. Chemical structures are reported in Figure 10.

**Figure 11:**
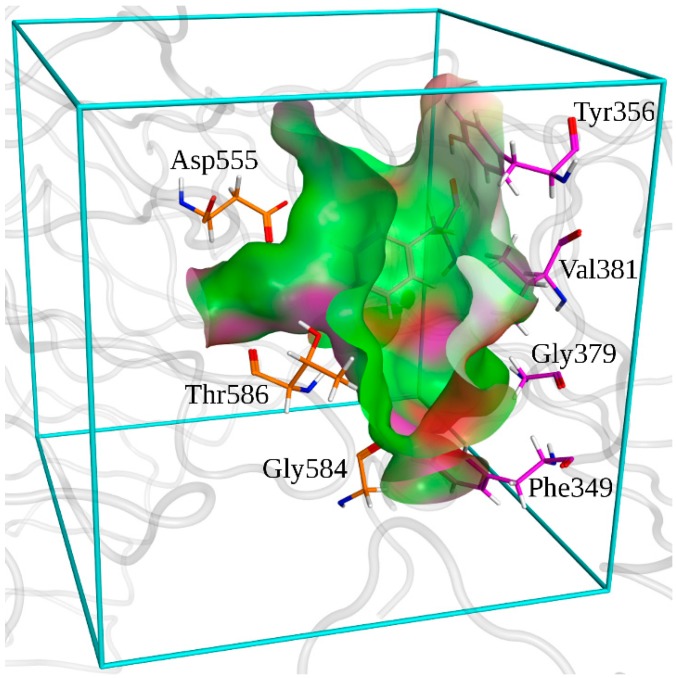
Surface of the binding pocket of TLR7. Hydrophobic zones are depicted in green. Polar zones are depicted in purple. Zones exposed to the solvent are depicted in red. Docking box used in Autodock is depicted in cyan. Residues that are conserved among TLR7 and TLR8 are depicted in orange for the first monomer and in purple for the second one.

The minimized holo-structure of TLR7 was used for the docking; the binding zone of TLR7 was determined as lying around residue Asp^555^ by previous studies [37,40] (Figure 11).

Autodock 4.2 program [57] and the Lamarckian genetic algorithm [58] were employed for the automated docking of small molecules into the zone delimited by the docking box (Figure 2); The merging of the charges of the nonpolar hydrogens and the computing of Gasteiger charges [59] for the receptor structure were performed using AutodockTools 4.2 [57]. Based on the knowledge of the binding pocket location stated by previous works [37,40] a cubic docking box of 56 points in x, y, and z directions with a grid spacing of 0.375 Å was designed. All the ligand structures were processed using LigPrep 2.8 [60] in order to obtain different protonation states at pH 5.5, tautomers and ring conformations. The set of parameters was chosen by docking R-848 (Resiquimod) in TLR8 crystallographic structure (Protein Data Bank id: 3W3N) and comparing the results of different sets with the experimental conformation; the best set was chosen as the one that allowed the reproduction of the conformation that was closest to the crystallographic one (PDB id: 3W3N) (Figure S1). The solution of the best set was compared also with the result of the MOE docking induced fit protocol [44], with a difference in RMSD value below 2 Å between the two solutions (Figure S2); this additional step was useful not only to verify if by using a different docking technique a similar solution was reached, but also to assess if with the introduction of a grade of flexibility (in the residues side chains trapped in the binding pocket), the resulting best pose is different from the one obtained with Autodock, which totally neglects the flexibility of the target. Parameters for MOE protocol were as following:

- 1000 initial poses of the small molecules were generated in the binding pocket;
- the reference position was taken as the position of the CL097 molecule obtained from the homology modeling process, and the conformation were created using the Triangle Matcher algorithm [61,62], which generates poses by aligning ligand triplets of atoms on triplets of alpha spheres dummies;
- the ranking of this first set of poses was made by using the London scoring function [44] that estimates the binding energy as [63]:
  (1) $${\text{ΔG}}_{\text{LdG}}{\text{= c + E}}_{\text{flex}}\text{ + }\sum_{\text{hbonds}}{\text{c}}_{\text{hb}}{\text{f}}_{\text{hb}}\text{ + }\sum_{\text{metal-lig}}{\text{c}}_{\text{m}}{\text{f}}_{\text{m}}\text{ + }\sum_{\text{i}}{\text{ΔD}}_{\text{i}}$$

In Equation (1), ${\text{c}}_{\text{hb}}$, ${\text{c}}_{\text{m}}$ and c are empirical parameters extracted from a set of 400 complexes, ${\text{E}}_{\text{flex}}$ is an estimation of the loss of the conformational entropy due to the binding, ${\text{f}}_{\text{hb}}$ and ${\text{f}}_{\text{m}}$ are measures of the geometric imperfections of the hydrogen bonds and metal-ligand interactions and ${\text{ΔD}}_{\text{i}}$ is the desolvation contribution modeled by using a volume integral London dispersion [47]. Based on the London scores, the thirty best conformations were selected from the initial set and energetically minimized using 1000 steps of Conjugate Gradients method with the Generalized Born implicit solvation method for the model of water. The induced fit protocol also allows consideration in the minimization step of the flexibility of the side chains within a certain distance from the ligand. In this work all the side chains within 5 Å of the molecule were considered as flexible with a weak restraint to the initial position. The all-atoms MMff94x [64,65] force field was used for the parameters of both the protein and drug atoms. The conformations obtained from these minimization steps were then scored with the GBVI/WSA scoring function [47,63]; this method is computationally more expensive than the Autodock one, as it calculates the free energy of binding as

(2) $${\text{ΔG}}_{\text{exp}}\text{= c + α[}\frac{\text{2}}{\text{3}}{\text{(ΔE}}_{\text{c}}{\text{ + ΔE}}_{\text{s}}{\text{) + ΔE}}_{\text{vdw}}{\text{ + βΔSA}}_{\text{w}}\text{]}$$

where c is the loss of the roto-translational entropy due to the binding, α and β are force field-depending experimental parameters, ${\text{ΔE}}_{\text{c}}$ is the Coulombic electrostatic term, ${\text{ΔE}}_{\text{s}}$ is the solvation energy referring to the General Born/Volume Integration model, ${\text{ΔE}}_{\text{vdw}}$ is the Van Der Walls term and ${\text{ΔSA}}_{\text{w}}$ is the surface area weighted by exposure. The ten best ranked conformations were conserved and the best one was used for the comparison with the Autodock one: the two conformations were loaded and then superimposed in MOE for the structural analysis. The set of parameters constitutes in an initial population of 300 random conformations, a maximal number of energetic evaluations of 25 million and a maximal number of generations of 27 thousand; the probability of performing a local search on an individual was kept at the default value of 0.06; one top individual was kept as a survivor for the next generation; the rates of gene mutation and crossover were set respectively to 0.02 and 0.8; 300 Solis-Wets local search iterations were performed for each generation; the algorithm was run one hundred independent times for every compound. The configuration files for each molecule were automatically generated using Raccoon [66]. Desolvation and electrostatic maps in the binding domain for each atom type were pre-calculated using Autogrid 4.2. Autodock united-atom scoring function was used to calculate the binding energies [67]. Docking results were grouped using the RMSD–based clustering as implemented in Autodock, with a cluster tolerance of 2 Å. The best conformer for each ligand was chosen as the pose with the lowest binding energy in the most populated cluster. Interactions with the receptor were plotted with Ligand Interactions Diagram tool in MOE [68].

Binding energies from experimental EC_50_ values were calculated for R-848, 52455, 52542 and 52763 using the following equation:

(3) $${\text{ΔG}}_{\text{exp}}{\text{=RTln(EC}}_{\text{50}}\text{)}$$

where R is the gas constant equal to 0.001987 kcal/mol/K, T is equal to 300 K and the ${\text{EC}}_{\text{50}}$ values are experimental data [30].

### 4.3. Molecular Dynamics

Explicit hydrogens were reintroduced to the docked complexes using Protonate 3D. Missing parameters for the small molecules were assigned using antechamber; ff12SB and GAFF parameters were assigned for the protein and for the ligands, respectively with leap; the systems were inserted into an octahedral box with a buffer of 12 Å of TIP3P water molecules and neutralized with chlorine ions. in pmemd.cuda, two stages of minimization were performed: the first step was run with a restraint of 500 Kcal/mol/Å^2^ on the complexes, in order to allow the relaxation of the water molecules using 1000 steps of Steepest Descent method followed by 1000 steps of Conjugate Gradients method. Then, the restraints were removed and the whole system was minimized with 2000 steps of Steepest Descent followed by 3000 of Conjugate Gradients. The system was then heated up to 310 K in 100 ps using the Langevin thermostat with a time collision frequency of 2 ps and a time step of integration of 0.5 fs. Target temperature was reached after 150,000 steps and maintained for the last 50,000 in this step. An NVT ensemble was simulated, with a restraint of 2 kcal/mol/Å^2^ put on the protein backbone and on the heavy atoms of the small molecule. The restraints were slowly removed in four phases of 50 ps each with a step of 0.5 kcal/mol/Å^2^ and a production simulation for an NPT system was then performed for 10 ns with a time step of 2 fs at 1 atm, using Berendsen barostat; bonds involving hydrogens were blocked using the SHAKE algorithm. The trends of the RMSD between the protein backbone, the ligand and the residues included in the binding zone of the simulated systems and the initial conformations were calculated using AmberTools12 ptraj.

A 30 ns long Molecular Dynamics simulation of the solvated receptor without any ligand was performed; ff12SB parameters were assigned for the protein with tleap. The system was inserted into an octahedral box with a buffer of 12 Å of TIP3P water molecules and neutralized with 43 chlorine ions. In pmemd.cuda, two stages of minimization were performed: the first step was run with a restraint of 500 kcal/mol/Å^2^ on the protein, in order to allow the relaxation of the water molecules using 1000 steps of Steepest Descent method followed by 1000 steps of Conjugate Gradients method. Then, the restraints were removed and the whole system was minimized with 2000 steps of Steepest Descent followed by 3000 of Conjugate Gradients; the system was heated up to 310 K in 100 ps using the Langevin thermostat [69] with a time collision frequency of 2 ps and a time step of integration of 0.5 fs. The target temperature was reached after 150,000 steps and maintained for the last 50,000 in this step. An NVT ensemble was simulated, with a restraint of 2 kcal/mol/Å^2^ on the protein backbone. Afterwards the restraints were slowly removed in four phases of 50 ps each, with a step of 0.5 kcal/mol/Å^2^ and a production simulation (NPT ensemble) was run for 30 ns with a time step of 2 fs at 1 atm, using Berendsen barostat [70]. Bonds involving hydrogens were kept fixed using the SHAKE algorithm [71]. The trend of the RMSD between the backbone of the simulated system and the initial conformation was evaluated using AmberTools12 ptraj. The trend of the total energy of the system was also evaluated.

### 4.4. Free Energy Estimates

Binding free energies were calculated using the script MMPBSA.py [72] in AmberTools12. The free energy (or binding energy) is the most important measurable quantity in drug design works, as it indicates the affinity between the ligand and the target; the lower the value for this energy the better is the affinity between the ligand and the target.

In this work the Generalized Born model of solvation (MMGBSA) [73,74] was used in order to calculate relative free energies of binding, as it permits the establishment of a good balance between the speed of the calculations and the correctness of the rank between the investigated compounds.

The free energy is calculated with the equation [75]:

(4) $${\text{ΔG}}_{\text{bind,solv}}{\text{ = ΔG}}_{\text{bind,vacuum}}{\text{ + ΔG}}_{\text{solv,complex}}{\text{ - ΔG}}_{\text{solv,ligand}}{\text{ - ΔG}}_{\text{solv,receptor}}$$

The in-vacuum contribution estimates the non-bonded interactions (Van der Waals, hydrogen bonds, Coulomb) between the two molecules and it can include the entropic term modeling the loss of entropy due to the bond, through the relation:

(5) $${\text{ΔG}}_{\text{bind,vacuum}}{\text{ = ΔG}}_{\text{nonbond}}\text{ - TΔS}$$

It is possible to calculate the entropy contribution by performing normal mode (translation, rotation and vibration modes) [76,77] or quasi-harmonic [78] analysis; however, this step is quite expensive in terms of computational cost and it is not necessary if the goal is to estimate binding affinities with a common target, where the entropic contribution can be considered as constant. In this case a relative final rank of compounds instead of an absolute one is acceptable.

As is possible to see from the free energy equation the energy of binding takes into account also the solvation contribution; the Generalized Born (GB) method is indeed a way to treat the modeling of the aqueous environment surrounding the systems by using an implicit solvent model, that is the replacement of explicit water molecules with an infinite continuum medium with the dielectric properties [73]. This approach approximates the solvent electrostatic contribution in the Poisson-Boltzmann (PB) model [74]. In the GB method the solvation contribution is modeled as

(6) $${\text{ΔG}}_{\text{solv}}{\text{ = ΔG}}_{\text{el}}{\text{ + ΔG}}_{\text{nonpolar}}$$

decomposed in an electrostatic and a non-electrostatic part; the first term is the free energy obtained by removing the charges from the system in vacuum and re-adding them in a solvated environment and it is modeled with the analytical generalized Born approximation [79] as

(7) $${\text{ΔG}}_{\text{el}}\text{= -}\frac{\text{1}}{\text{2}}\sum_{\text{ij}}\frac{{\text{q}}_{\text{i}}{\text{q}}_{\text{j}}}{{\text{f}}^{\text{GB}}{\text{(r}}_{\text{ij}}{\text{,R}}_{\text{i}}{\text{,R}}_{\text{j}}\text{)}}\text{(1 - }\frac{{\text{e}}_{\text{ij}}^{{\text{-kf}}^{\text{GB}}}}{{\text{ε}}_{\text{w}}}\text{)}$$

where the i^th^ atom has radius ${\text{ρ}}_{\text{i}}$ and its charge ${\text{q}}_{\text{i}}$, and it is considered as filled by a medium with a dielectric constant of 1, whereas it is surrounded by a continuum medium with an elevate dielectric constant ${\text{ε}}_{\text{w}}$ (80 at 300 K for water); ${\text{r}}_{\text{ij}}$ is the distance between atoms i and j, ${\text{R}}_{\text{i}}$ is the effective Born radius of each atom, indicating the level of burial inside the molecule and ${\text{f}}^{\text{GB}}$ is a smooth function [79,80,81]. ${\text{ΔG}}_{\text{nonpolar}}$ is the energy associated to the solvated molecule when it is not charged, usually considered as proportional to the solvent accessible surface area (SASA) as:

(8) $${\text{ΔG}}_{\text{nonpolar}}\text{= γA(x)}$$

where γ is the surface tension and A(x) is the structure area accessible to the solvent of the conformation X. An empirical term to model the hydrophobic contributions is often added to the solvation free energy. The energy of binding is calculated on each of a series of snapshots taken from a Molecular Dynamics simulation of the complex and then the values are averaged to obtain the final result. The GB method is widely used in the field of Molecular Dynamics [82] thanks to its computational efficiency; moreover, the ranking performances obtained in drug design works are comparable with the ones of the MMPBSA method, more computationally expensive and slower than the MMGBSA [83,84]. These two techniques also permit separate calculation of the interactions between every pair of residues; this tool is particularly important in rational drug design, where the study of the interactions between each of the residues in the binding pocket and the ligand allows the determination of the residues that are most important in ligand binding [72]. The igb flag specifies the GB model used for the calculations and it was set as igb = 5, referring to a modified model developed by Onufriev *et al.* in which Born radii are rescaled [73].

Two hundred and fifty snapshots for each complex were evaluated from the last 5 ns of simulation in order to calculate the relative binding free energies using the MMGBSA method without considering the entropic contribution; igb flag was set to 5 [73]. The pairwise per-residue decomposition of the binding energy was also performed for the two imidazoquinoline derivatives in addition to 1V209, 52455 and 52459, in order to show the residues that are most involved in ligand binding; residues within 5 Å from the molecules in the binding pocket were considered for this step.

## 5. Conclusions

The TLR7 is a promising pharmaceutical target for the treatment of several diseases, including cancer and viral pathologies; a major obstacle for the development of novel compounds targeting this membrane protein is however the lack of an experimental three dimensional structure. In this work we employed the homology modeling technique to build a high quality three all-atom structure of the TLR7, using as template the recently published experimental structure of TLR8. The TLR7 dimeric model has been then used as target for molecular docking and Molecular Dynamics simulations, in order to study the binding poses of two classes of TLR7 activators. Free energy estimations have been performed in the final step in order to determine the quantitative characteristics of the binding as well as the single contributions from the residues in the binding zone: the active compounds showed higher stabilities and binding affinities compared with the inactive ones, demonstrating the reliability of our model as a platform for virtual screening. In addition five residues within the binding pocket, namely Asp^555^, Ile^585^, Thr^586^, Phe^408^ and Leu^557^, have been found to contribute mainly to the interactions with the ligands. The accurate description of the binding mode of the TLR7 agonists as well as the features of the binding pocket that we present in this paper can be a starting point for the design and the development of novel compounds targeting the human innate immune system for the treatment of a wide range of diseases.

## Supplementary Materials

Supplementary materials can be accessed at: http://www.mdpi.com/1420-3049/20/05/8316/s1.
