# Supplementary material for: Structure Based Modeling of Small Molecules Binding to the TLR7 by Atomistic Level Simulations
Source: Molecules. 2015 May 8;20(5):8316–40. doi: 10.3390/molecules20058316 (PMC6272798; doi:10.3390/molecules20058316)
Supplement: Supplementary file 1 [file molecules-20-08316-s001.pdf]

Supplementary Materials

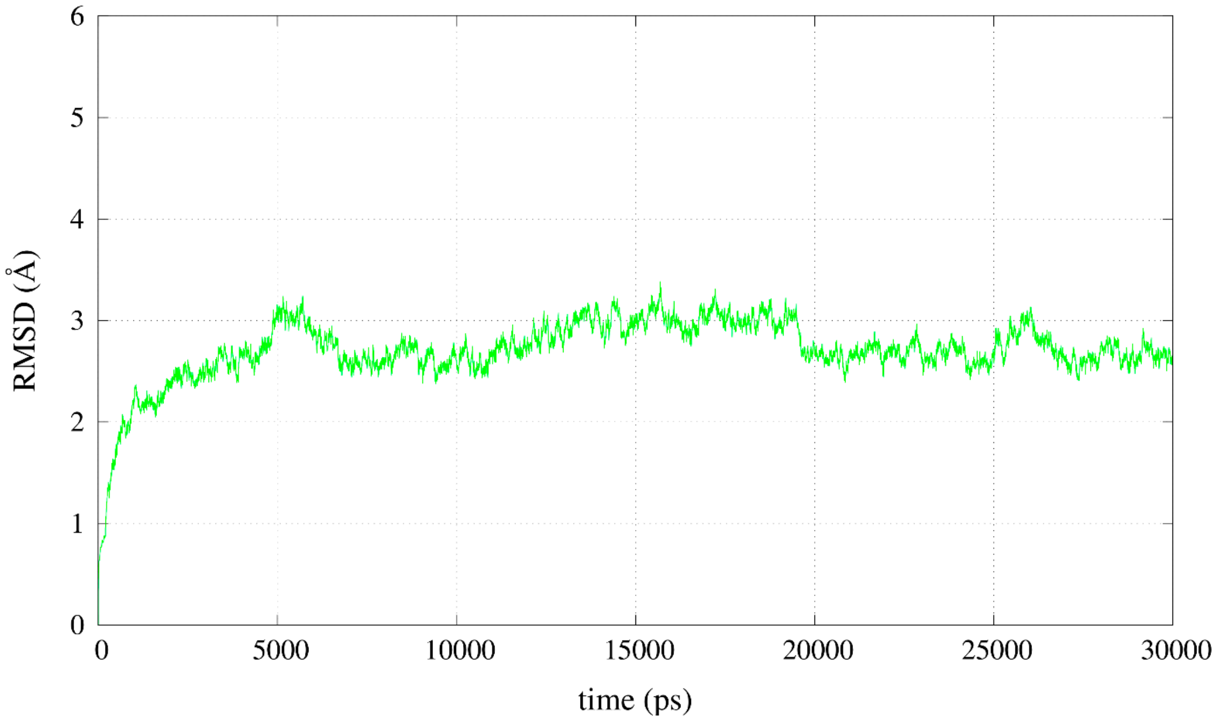

Figure S1. RMSD trend for the apo-structure of TLR7.

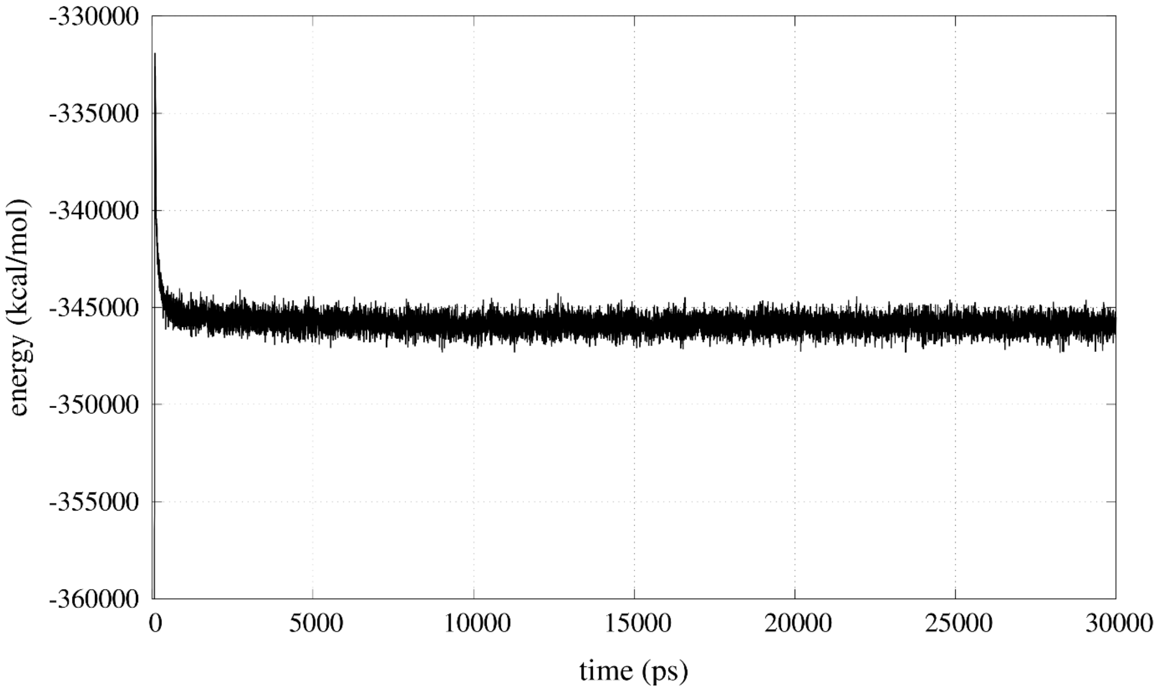

Figure S2. Total energy for the apo-structure of TLR7.

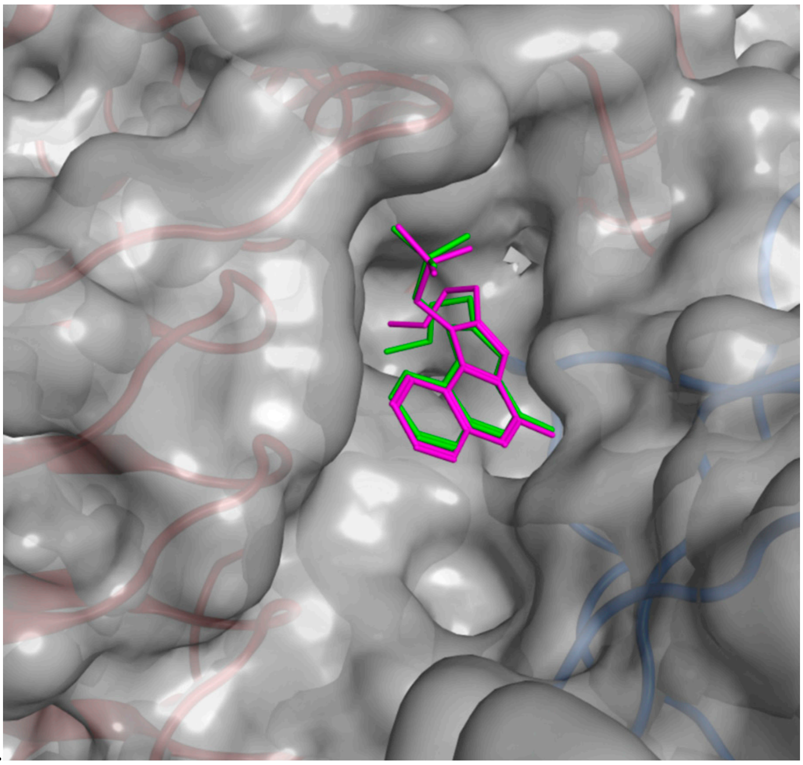

.

Figure S3. Superimposition of crystallographic and docked conformations of R-848 on TLR8. Experimental structure is represented in purple; docking result is depicted in green; pocket surface is represented in gray.

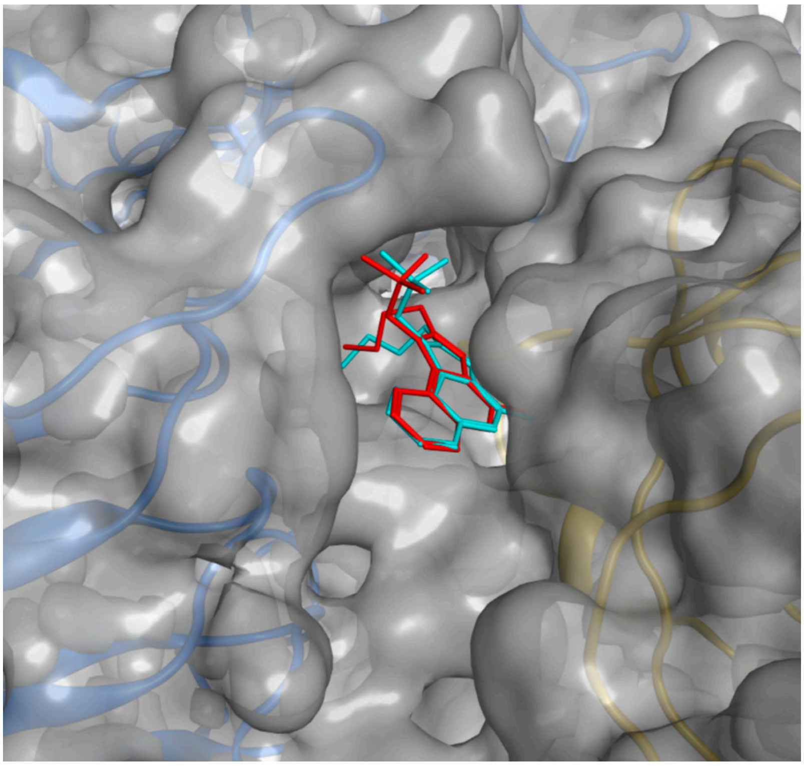

Figure S4. Superimposition of docked conformations of R-848 on TLR7 from Autodock and MOE. Autodock structure is represented in red; MOE result is depicted in cyan; pocket surface is represented in gray.

Table S1. Results from Autodock are compared with experimental binding energies.

Compound	Autodock			Experimental		
	Binding Energy (kcal/mol) [1]	Size of Cluster [2]	Ligand Efficiency [3]	EC_{50} (nM) [4]	SD (nM) [4]	Binding Energy (kcal/mol) [5]
R-837	−7.29	96	−0.41	n.a.	n.a.	n.a.
R-848	−7.79	56	−0.34	607	240	−8.53
1V209	−8.23	59	−0.32	n.a.	n.a.	n.a.
52455	−8.53	100	−0.37	103	12	−9.60
52457	−6.09	43	−0.28	-	-	-
52459	−7.93	100	−0.36	-	-	-
52542	−8.06	96	−0.35	353	96	−8.86
52587	−7.10	41	−0.32	-	-	-
52763	−8.15	99	−0.37	965	283	−8.26

SD refers to standard deviations. n.a. refers to unavailable data for agonists. - refers to compounds that did not show any activity on TLR7; [1] binding energy of the lowest energetic conformation in the most populated cluster; Autodock error is ~2.5 kcal/mol; [2] size of the most populated cluster (maximal value is 100); [3] calculated as binding energy/number of heavy atoms; [4] from [1]; [5] calculated as $RT\ln(EC_{50})$; see text for more details.

Table S2. Relative binding energies resulting from MMGBSA calculations over Molecular Dynamics trajectories.

Compound	Binding Energy (kcal/mol)	SD (kcal/mol)	Activity
R-837	−31.081	2.530	yes
R-848	−40.734	3.986	yes
1V209	−50.251	4.360	yes
52455	−41.136	3.971	yes
52457	−23.592	2.982	no
52459	−22.476	3.265	no
52542	−48.162	3.523	yes
52587	−21.117	4.062	no
52763	−35.550	3.996	yes

SD refers to standard deviations.

Table S3. Single residues contributions to MMGBSA binding energies of 1V209, R-837, R-848, 52455, 52459.

Residue	Binding Energy Contribution (kcal/mol)				
	**1V209**	**R-837**	**R-848**	**52455**	**52459**
aTHR532	−0.368	−0.129	−0.447	−0.134	−2.642
aASP555	−11.297	−2.259	−3.572	−9.212	−0.998
aLEU556	−0.165	−1.438	−0.969	−0.084	−0.142
aLEU557	−5.259	−4.818	−4.783	−4.457	−0.916
aTYR579	−0.548	−1.104	−0.337	−1.405	−0.074
aGLY584	−0.693	−0.269	−1.016	−1.006	−0.383
aILE585	−5.078	−4.426	−4.362	−4.926	−1.513
aTHR586	−6.570	−5.101	−5.693	−5.477	−0.347
bPHE349	−0.869	−0.052	−0.763	−1.004	−0.030
bPHE351	−2.428	−2.125	−3.268	−2.514	−0.221
bVAL355	−1.422	−0.408	−1.561	−0.990	−2.050
bTYR356	−4.137	−0.644	−2.259	−2.771	−4.980
bGLY379	−0.676	−0.082	−0.859	−0.829	−0.051
bVAL381	−2.350	−1.340	−2.586	−2.573	−1.112
bTHR406	−0.833	−0.124	−0.837	−1.011	−0.095
bASN407	−0.273	−0.109	−0.364	−0.288	−0.115
bPHE408	−6.264	−2.719	−5.596	−5.303	−1.897
bLYS432	−1.908	−0.267	−1.958	−2.225	−1.117

a and b suffixes in the residues column refer respectively to the first or the second monomer of TLR7.

References

1. Forsbach, A.; Müller, C.; Montino, C.; Kritzler, A.; Nguyen, T.; Weeratna, R.; Jurk, M.; Vollmer, J. Negative regulation of the type I interferon signaling pathway by synthetic Toll-like receptor 7 ligands. *J. Interferon Cytokine Res.* **2012**, *32*, 254–268.
